# Analysis of an Advisor–Advisee Relationship: An Exploratory Study of the Area of Exact and Earth Sciences in Brazil

**DOI:** 10.1371/journal.pone.0129065

**Published:** 2015-05-26

**Authors:** Esteban F. Tuesta, Karina V. Delgado, Rogério Mugnaini, Luciano A. Digiampietri, Jesús P. Mena-Chalco, José J. Pérez-Alcázar

**Affiliations:** 1 Information Systems Program/School of Arts, Sciences and Humanities (EACH), University of São Paulo, São Paulo, Brazil; 2 School of Communication and Arts (ECA), University of São Paulo, São Paulo, Brazil; 3 Center for Mathematics, Computation and Cognition (CMCC), Federal University of ABC, São Paulo, Brazil; Northwestern University, UNITED STATES

## Abstract

Scientific collaboration has been studied by researchers for decades. Several approaches have been adopted to address the question of how collaboration has evolved in terms of publication output, numbers of coauthors, and multidisciplinary trends. One particular type of collaboration that has received very little attention concerns advisor and advisee relationships. In this paper, we examine this relationship for the researchers who are involved in the area of Exact and Earth Sciences in Brazil and its eight subareas. These pairs are registered in the Lattes Platform that manages the individual curricula vitae of Brazilian researchers. The individual features of these academic researchers and their coauthoring relationships were investigated. We have found evidence that there exists positive correlation between time of advisor–advisee relationship with the advisee’s productivity. Additionally, there has been a gradual decline in advisor–advisee coauthoring over a number of years as measured by the Kulczynski index, which could be interpreted as decline of the dependence.

## Introduction

Brazil has been regarded as a developing country in the last few decades and has shown a robust growth in several areas, including scientific research. Funding by the Brazilian government has been of critical importance for the development of this area. The initial milestones of public investment in science were the establishment of agencies such as the Coordination for the Improvement of Higher Education Personnel (CAPES) and the Brazilian National Council for Scientific and Technological Development (CNPq) in the fifties [1] which give financial support and offer incentives to Brazilian post-graduate programs. Owing to the expansion and decentralization policies of the nineties, there was a significant growth of academic research output in the country [2].

Brazilian scientific policies employ a wide-ranging institutional system that covers the post-graduate programs across the country, and these are consolidated into a bulky triennial assessment procedure, which in 2013 brought together consultants to evaluate 3,342 post-graduate programs involving 65,235 researchers over a period of four weeks. Among the factors that are analyzed as in related work [3], the scientific productivity is measured by the number of publications in journals of an officially recognized scientific standard, and thus it is expected that the PhD student is inserted in a research environment where results are determined by the publication of papers. Data about PhD graduate programs in Brazil show that 105,399 PhD degrees were awarded between 1981 and 2010 (estatico.cnpq.br/painelLattes/evolucaoformacao).

Participating in research is the most important activity of PhD students, which may be measured through the publication outputs. According to Pinheiro et al (2014) students develop skills and “know-how” during the doctoral period that enable them to train the process of writing and submitting papers [4]. One of their findings is concerned to the positive impact of the collaboration with the advisor to the PhD graduate publications. Different from this, in this work it is studied the influence of the collaboration with the advisor not only for the period of the graduation, but also after this period. The novelty of our approach is to measure the temporal evolution of the dependence index between advisor and advisee. The analyzed data corresponds to a group of PhD researchers in the Exact and Earth Sciences Area registered in the Lattes Platform (a comprehensive platform of curricula vitae of researchers affiliated or related to Brazilian institutions). The advantage of using data obtained from this Platform, is that the advisor-advisee relationship is fully identified by this system and allows large scale bibliometric studies.

## Related Work

A recent study of the productivity of post-graduate students from the Institute of Energy and Nuclear Research post-graduate program, show that, in its more than 30 years of existence, the time period to publish papers related to that spent on a thesis has declined in the last 10 years [5] (this period is calculated as the mean of the difference between the years of publication of the papers and the year of the thesis defense) and in recent decades, there has been an increasing number of papers derived from each thesis.

The burgeoning number of post-graduate students involved in scientific communication has been the subject of studies in many countries that have been discussing the role of master´s and doctorate courses in the research field, among them: France [6], Croatia [7], Brazil [8, 9], and Peru [10]—most of them in areas of Health Science—and in the United States [11–13], United Kingdom [14] and Canada [15]—in several areas. Generally, these studies consist of collecting theses together with the authors’ names and searching for their scientific productions in the databases of journals. Some of them use the name of the supervisor as a criterion for deciding if the paper originated from a doctoral research study. The first large-scale analysis of Canadian doctoral student research effort is provided by [15] that uses automatic and manual validation to solve homonym’s problems. Another factor is the time of publication that is required to collect published papers: for example, 5 years before and after the thesis defense [5].

There are also qualitative studies that analyze interpersonal relationships between advisor-advisee (A-A) and consider them to be essential to the success of the PhD project. The instrument used to measure this relationship is, generally, a questionnaire [16, 17]. Other studies [18–21] also stress the importance of this relationship. Some studies have also examined this relationship and state that this collaboration positively influence the publication output of PhD students before and after graduation [4, 22, 23]. The impact of scholarships in the publication output after the PhD graduation has been assessed by [3, 24].

Recently, the A-A academic relationship in the area of computer science was investigated to infer the existence of this relationship by means of data mining tools and computational learning [25–28].

## Materials and Methods

### Data Description and Notation

The data in this paper were retrieved from the Lattes platform (lattes.cnpq.br). According to the information provided by this platform, there were 105,399 doctors who graduated until 2011, 10,953 of them registered the Exact and Earth Sciences as their main doctoral area.

The curricula were downloaded following the methodology used by [29] and stored in a relational database following the strategy used by [30]. The processed data is related with the following: academic degrees, supervision, gender, and publications in peer-reviewed journals. The data analyzed are journal papers per year, gender, the year of beginning the PhD and the year of PhD graduation. Furthermore, in the case of each advisor-advisee pair, we obtained the scientific output that was published through the collaboration between the advisor and the advisee. This study examined the number of publications, the year of beginning the PhD, and the year of PhD graduation that were carried out until 2011.

The supervisory relationships were established through a double check between the analyzed curricula; i.e. we only regarded those that appeared in the curricula of the advisee and advisor as forming a supervisory relationship.

An approximate string matching algorithm was employed to determine the coauthoring relationship by comparing the publication titles, the names of the journals, and the coauthors’ lists.

Let Ω be the set of advisors, and Θ be the set of advisees. The number of advisors is ∣Ω∣ and the number of advisees is ∣Θ∣, where ∣.∣ is the cardinality.

In the sample set, we found ∣Θ∣ = 8,250 (15 advisees had more than one doctoral degree or else registered the co-advisor as an advisor), 5,429 advisees are men and 2,821 are women. We also found ∣Ω∣ = 3,184, 2,513 advisors are male and 671 female. ∣Θ∩Ω∣ = 481, i.e. 481 researchers played the roles of advisee and advisor.

Given an author *i*, the total research output *O*_*i*_ is composed by research outputs *o*_*ix*_ authored or co-authored by i: *O*_*i*_ = {*o*_*i*1_, *o*_*i*2_, ⋯, *o*_*in*_}. The number of articles *p*_*i*_ produced by author *i* is *p*_*i*_ = ∣*O*_*i*_∣. Given two authors *i* and *j*, their joint research output *O*_*ij*_ is defined as a set composed by outputs *o*_*ijx*_ co-authored at least by both *i* and *j*: *O*_*ij*_ = {*o*_*ij*1_, *o*_*ij*2_, ⋯, *o*_*ijn*_}. The number of articles *p*_*ij*_ produced by both author *i* and *j* is *p*_*ij*_ = ∣*O*_*ij*_∣.

From the whole research output, only journal papers were included in the analysis. The sum of joint publication between advisee and advisor is ∑_*i* ∈ Θ_ *p*_*ij*_ = 27,454, where *j* ∈ Ω is the corresponding advisor of each *i*. The total number of advisors’ outputs is ∑_*i* ∈ Ω_ *p*_*i*_ = 164,852 and the total number of advisees’ outputs is ∑_*i* ∈ Θ_ *p*_*i*_ = 93,953.

The Exact and Earth Sciences Area (Exact Sciences for short) in the Brazilian National Council for Scientific and Technological Development (CNPq) database comprises eight subareas: Astronomy, Chemistry, Computer Science, Earth Sciences, Mathematics, Oceanography, Physics and Probability and Statistics. All the features of the Exact Sciences were also collected for these subareas. Thus, the analyses were performed for the complete dataset and for each subarea together with the three decades called: D1 = 1981–1990, D2 = 1991–2000 and D3 = 2001–2010 considering the year of beginning the PhD.

Given an advisee *i*, let *b*_*i*_ the year of beginning the PhD and *g*_*i*_ the year of PhD graduation. The year of the first and last publication for an author *i* are represented by $y_{i}^{f}$ and $y_{i}^{l}$, respectively. Without loss of generality, let $y_{ij}^{f}$ be the year of the first output of the author *i* with or without a coauthor *j*.

### Method

The approach used in this work to address the research questions consist of dividing our analysis in three blocks:

1. Study about time relationship between PhD graduate and advisor
  - Has the time needed to obtain a PhD degree changed in recent decades? The time period to complete a PhD degree $T_{i}^{C}=g_{i}-b_{i}$ has been calculated for each advisee *i*.
  - Are there differences between the distributions of the time until the first publication with/without the advisor? We have defined the elapsed time until the first publication of PhD graduate with his advisor with respect to the year of the PhD graduation as $z_{i}=y_{ij}^{f}-g_{i}$, where *i* is the advisee and *j* his advisor. Thus, negative (positive) values of *z*_*i*_ indicate the first output was published before(after) the year of PhD graduation. Similarly, we have defined $z_{i}^{*}$ as the elapsed time until the first publication without the advisor. We have observed the concentration and skewness of *z*_*i*_ and $z_{i}^{*}$.
  - Has it been possible that the duration of the period of coauthorship extrapolates from the formal period of supervision? Two approaches have been adopted to address this question. The first takes account of the time relationship $T_{i}^{R}$ of PhD graduate *i* with his advisor which has been defined as the maximum time relationship from the beginning of the PhD, i.e. $T_{i}^{R}=max(y_{ij}^{l},g_{i})-b_{i}$. The second only considers the pairs who have published at least one work after the end of the formal period of their academic relationship, i.e. $y_{ij}^{l}>g_{i}$. Thus, the extrapolated time has been defined as $T_{i}^{E}=y_{ij}^{l}-g_{i}$. In both cases *i* is the PhD graduate and *j* is the advisor. In addition, to make a comparison between different decades, we have calculated for each decade and subarea the ratio of extrapolation $v_{i}=T_{i}^{E}/(y_{ij}^{l}-b_{i})$, which allows to normalize $T_{i}^{E}$ in the interval [0, 1] and to reduce the cumulative advantage.
2. Study about research output
  - Has the proportion of advisees who have never published an article with their corresponding advisors fallen during the time or decade in question? The number of pairs that has not published with their advisors (pairs with *p*_*ij*_ = 0) was calculated for each decade.
  - What is the proportion between the total number of joint publications and the total number of the advisee’s publications? For each year *y* we compute *q*_*y*_ = ∑_*i* ∈ Θ_ *p*_*ij*_/∑_*i* ∈ Θ_ *p*_*i*_, where *j* is the advisor of *i*. Note that the total number of publications *p*_*i*_ also includes the publications with only one author.
3. Study about time relationship versus research output
  - Does the time of the relationship correlate with the total number of advisee’s publications? We compute the Spearman correlation coefficient between $T_{i}^{R}$ and *p*_*i*_. This coefficient is a non-parametric measure which calculates the strength of association between two variables. This differs from the Pearson correlation coefficient since it does not require normality assumption or linearity of the association. In the correlation analysis, we have used 95% as a significance threshold.
  - Has the A-A relationship measured by the relative joint production decreased during the time? We adapted the Kulczynski index [25] to measure the advisor-advisee coauthoring.

The Kulczynski Coefficient [25, 28] was used in data mining literature to discover the advisor-advisee relationship in a database of published papers. Wang et al. [25] aggregated the time factor and defined the Imbalance Ratio for computing the likelihood of one researcher i being an advisor of j; the candidate with the maximum likelihood is chosen by this method. In this study, it has not been necessary to discover this relationship because it has been recovered from the curriculum. We have taken this index for two reasons: (i) it is possible to assess the temporal relationship between advisors and their respective advisees; this has been carried out by taking the year of the first joint publication $y_{ij}^{f}$ as the beginning of a commitment, and (ii) the Kulczynski index has an important null-invariance property, i.e. its value is free from the influence of pairs that do not contain any of the elements being examined. As in [25, 28] we have defined the Kulczynski index for the pair *ij* (i advisor, j advisee) from $y_{ij}^{f}$ until year *t* as follows:

$$\begin{array}{c} kulc_{ij}^{t}=\sum_{k\leq t}\frac{p_{ij}^{k}}{2}\times (\frac{1}{\sum_{k\leq t}p_{i}^{k}}+\frac{1}{\sum_{k\leq t}p_{j}^{k}}), \end{array}$$ (1)

It should be observed that the Kulczynski index has been defined as 0 when there are no joint publications and has a maximum value of 1 when *O*_*i*_ = *O*_*j*_. Note that while the Kulczynski coefficient is used to study the A-A relationship using the relative joint production during the time, the correlation coefficient r is used to analyze the static influence of the time-relationship in the number of the advisee’s publications.

## Results

Table 1 contains information about the number of researchers, number of A-A pairs for each subarea and number of publications. It should be noted that the sum of the number of researchers was slightly different since some of them were registered in more than one subarea, and others have not registered in any subarea.

**Table 1:** Number of researchers, number of advisor-advisee pairs and number of publications for each subarea.

	No. of researchers	No. of pairs	No. of advisor pub.	No. of advisees pub.	No. of A-A pub.	Total No. of pub.
Astronomy	175	117	3,610	1,496	588	4,760
Chemistry	3,187	2,523	57,757	34,595	11,575	83,410
Computer Sc.	1,742	1,325	10,880	6,769	1,946	16,450
Earth Sc.	1,634	1,169	20,151	10,407	2,300	28,500
Math	1,074	761	11,201	4,289	954	14,425
Oceanography	348	233	4,849	2,067	496	6,559
Physics	2,398	1,745	55,438	29,868	9,118	76,620
Prob. & Stat.	398	288	7,622	3,906	635	10,936
Total	10,953	8,265	164,852	93,953	27,454	234,426

A few researchers (175) referred to Astronomy as their main research subarea. We observed, for example, that for decade D1, there were only 14 joint publications. This is probably because there are few universities in Brazil that offer Astronomy as an undergraduate course. Most of the researchers in Astronomy in Brazil did Physics as an undergraduate course and continued their scientific career in Astronomy.

The results are divided into three blocks as defined in the Method Section.

### Measuring the time relationship

#### Time to obtain the PhD degree ($T_{i}^{C}$)

**Fig 1:**
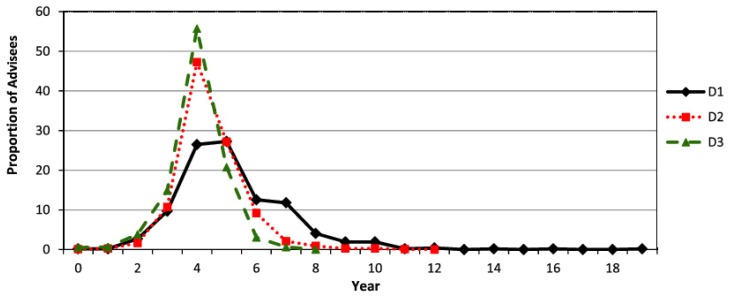
Frequency distribution of the number of years to obtain the PhD degree ($T_{i}^{C}$) for Exact Sciences by decades. (D1 = 1981–1990, D2 = 1991–2000 and D3 = 2001–2010).

We have observed that for almost all the subareas, the median time a student has obtained a doctorate (i.e. $T_{i}^{C}$) is 5 years for the decade D1 and reduced it to 4 in the decades D2 and D3. The frequency distribution of $T_{i}^{C}$ for the Exact Sciences is shown in Fig 1.

It has been observed a significant decrease in the coefficient of variation of the frequency distribution for the Exact Sciences area that are 37.08, 24.89 and 22.69 for decades D1, D2 and D3 respectively. Similarly for the Skewness, that changes from positive to negative with values 1.74, 1.07 and -0.5 for decades D1, D2 and D3 respectively. Additionally, we compare these distributions through the two sample Kolmogorov-Smirnov Test that shows a significant difference between decades (all with *p* < 0.01). The obtained statistics are 0.2077, 0.3628 and 0.1552 for the pairs (D1,D2), (D1,D3) and (D2,D3), respectively.

This behavior could be explained by the newly established rules of the CAPES and CNPq (agencies that coordinate the Brazilian post-graduate studies programs). They have established maximum period for PhD students to be awarded grants. Thus, as expected, the mean time to complete the PhD has been significantly reduced.

#### Time until the first publication (*z*_*i*_ and $z_{i}^{*}$)

It was found that throughout the entire time period all the subareas have a statistically significant reduction of the elapsed time until the first publication *z*_*i*_. To compute the difference between the distributions, it has been used the Kolmogorov-Smirnov test. The statistics for pairs (D1,D2) and (D1,D3) are *D* = 0.1261 and *D* = 0.2499, respectively, both with *p* < 0.01. Similarly, the result for the pair (D2, D3) is *D* = 0.1488 with *p* < 0.01. To test the null hypothesis of equality of the medians of these distributions, it has been used the Mann-Whitney test. For the pairs (D1,D2) and (D1,D3) the statistics are *W* = 496583.5 and *W* = 571644.5 both with *p* < 0.01. For the pair (D2,D3) the statistic W is 5018902.0 also with *p* < 0.01. It is easy to see that the largest proportion of the first publications of PhD graduate is concentrated on the supervised period, regardless of whether it was carried out with or without an advisor.

In particular, Astronomy, Chemistry, and Physics have shown over a period of decades that less time is required for the first publication. In the decade D1 most of the advisees produced their first works at the same time as at the end of the doctoral period; in the decade D3 this time is approximately two years earlier (data not shown in the manuscript). Mathematics showed the longest period; in the decade D1 the mean was 4 years after the finish of the PhD and in the decade D3 it was the same period as at the end of the doctoral period (data not shown).

**Fig 2:**
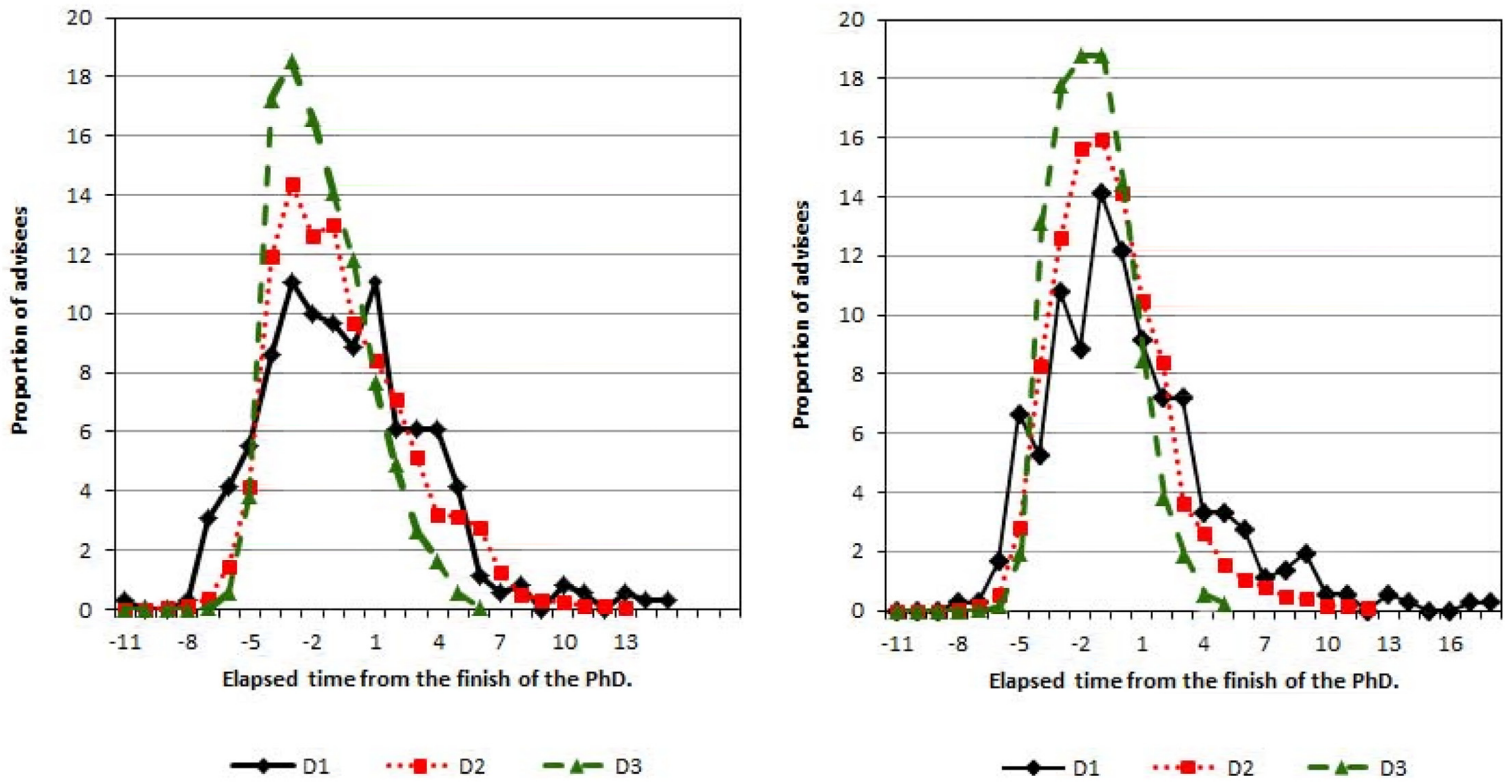
Distribution of the time to the first publication with and without the advisor (*z*_*i*_ and $z_{i}^{*}$) for Exact Sciences.

In Fig 2, the time to the first publication for the Exact Sciences by decade is represented. It can be seen that the distributions are invariably more concentrated before the finish of PhD. In addition, it can be noted that the skewness of the distributions is approximating zero.

The entire time period for the Exact Sciences has also been considered, and it was observed that the skewness value for the time until the first publication without the advisor ($z_{i}^{*}$) is positive (1.02) and less than the positive value of the skewness (1.41) for the time up to the first publication with the advisor (*z*_*i*_). As in the last analysis, we noted that in Astronomy, Chemistry and Physics subareas *z*_*i*_ and $z_{i}^{*}$ are less than in the other subareas. Mathematics is the subarea in which these times are the longest. For the Exact Sciences the mean time for *z*_*i*_ is near a year before the end of the PhD (-0.94) with a standard deviation of 2.59 years (here account has not been taken of individuals who have not published with their advisors) and on average, $z_{i}^{*}$ appears to be approximately the same year (-0.91) as *z*_*i*_ with a standard deviation of 3.14 years.

#### Time relationship between PhD graduate and his advisor ($T_{i}^{R}$) and the ratio of extrapolation (*v*_*i*_)

For Exact Sciences and for the entire period the mean of $T_{i}^{R}$ is about 6.07 years and is higher in approximately three years than the year of the first joint work (i.e. than $y_{ij}^{f}$ where *j* is the advisor). For the whole group and respective subareas, the median of $T_{i}^{R}$ is ≥ 5 years and in 75% of the pairs the median of $T_{i}^{R}$ is ≤ 7 years. In Chemistry and Physics, the mean and median are greater by about a year than in the other subareas and the third quartile of this distribution has also shown a greater value, which suggests that there is a close relationship between these pairs. Astronomy showed a singular behavior and high degree of variability because of the small number of researchers involved in this subject-area.

The mean of $T_{i}^{R}$ for the Exact Sciences, (when all the periods under study are taken into account), extends to 6.07 years and if we take the last decade, most of the students have completed their doctoral period in four years. Thus, it can be inferred from them that the duration of the coauthoring between advisor and advisee extrapolates the formal period of supervision in approximately 2 years. It should be noted that the suggested time that the Brazilian financial agencies believe is necessary to complete the PhD project is four years, and they have encouraged research independence, i.e. for scientific research to be carried out without the cooperation of the advisor after the thesis defense.

The ratio of extrapolation (*v*_*i*_) for Chemistry and Physics are greater than other subareas for each decade. That shows that Chemistry and Physics are the groups that actively collaborate over decades.

### Measuring the research output

#### Advisees without A-A publications

We observed that the A-A partnership has not always been productive since 2,312 PhD students have not published any material with their advisors (approximately 28% of the considered dataset) with the caveat that 68 of them had A-A publications before the start of the PhD and some have works without the advisor or that they have published at conferences or even in book form.

**Fig 3:**
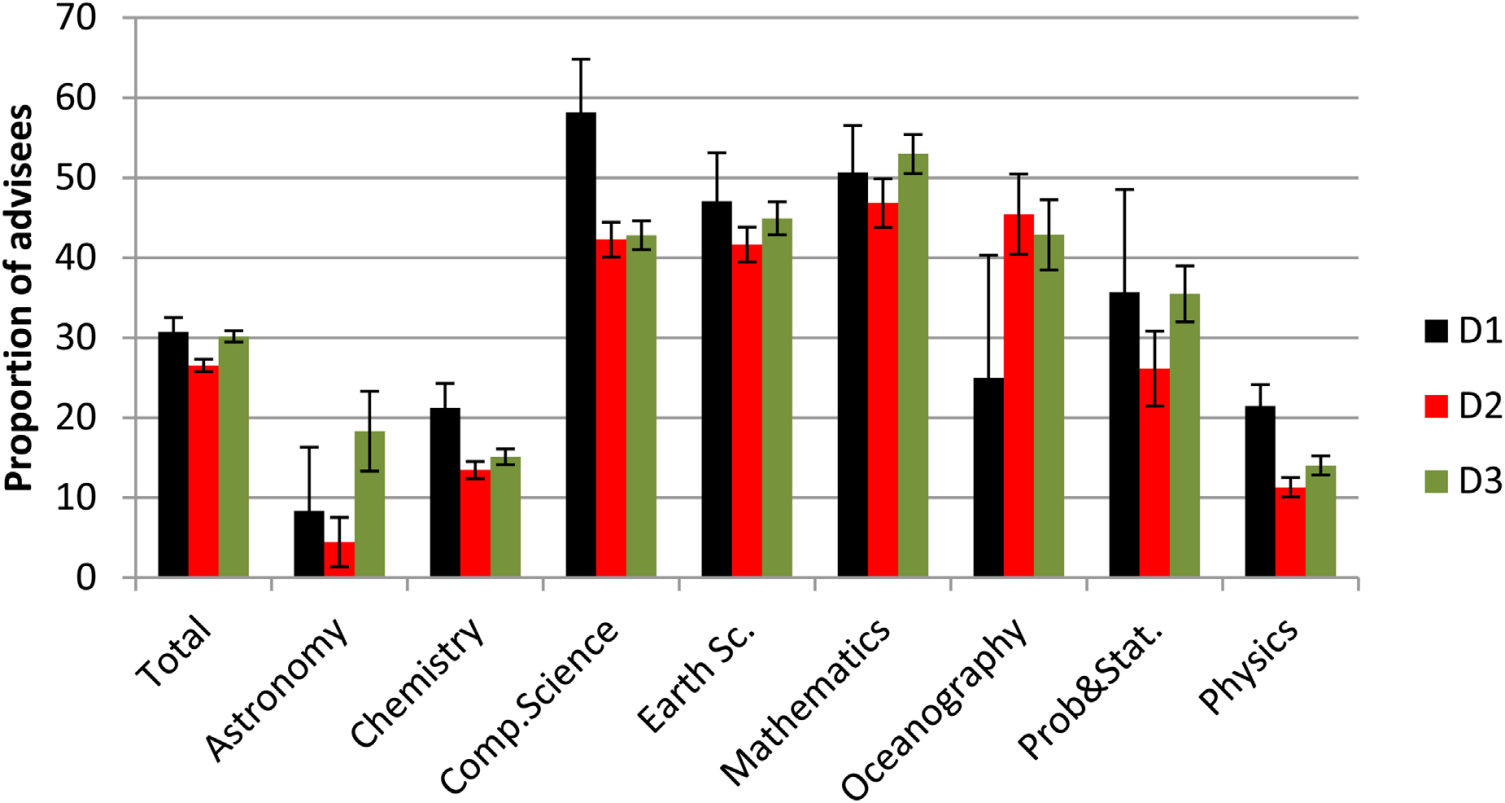
Proportion of advisees without A-A publications by decades.

The percentage of advisees who have not published with their advisors in Astronomy, Chemistry and Physics is lower than in other subareas for all the decades (See Fig 3). These proportions are not influenced by the variability of the sample nor the number of pairs. It should be noted that there was a significant reduction in the already mentioned proportion from decade D1 to D3 in Physics and Computer Science. Also this reduction is not influenced by the variability of the sample. Despite the small number of pairs in the first years of D1 and the last years of D3, the null hypothesis of the random distribution of the ranks of these two decades has been rejected by the Wilcoxon Rank Sum test for Physics and Computer Science. Fot these two subareas, the statistics are *W* = 89 (*p* < 0.01) and *W* = 68 (*p* = 0.032), respectively. Also, the same null hypothesis has been tested for Chemistry and it has not been rejected; the statistic is *W* = 63.5 with p-value not significant (*p* = 0.163). In contrast, a different behavior is observed for Astronomy and Oceanography that can be explained by the small number of pairs for the D1 decade. Note that the standard deviation for these two subareas in the decade D1 is greater than the others.

Through a triennial evaluation procedure for the graduate programs, CAPES has laid down guidelines for the pairs to produce research results in the form of papers, conferences, books or patents. Thus, it was expected that the proportion of advisees that have not produced A-A publications would decline during the decades. However, this expected behavior was not observed in Mathematics that has the highest percentage of advisees without A-A publications.

#### Proportion between the A-A publications and the total number of advisee’s publications per year (*q*_*y*_)

**Fig 4:**
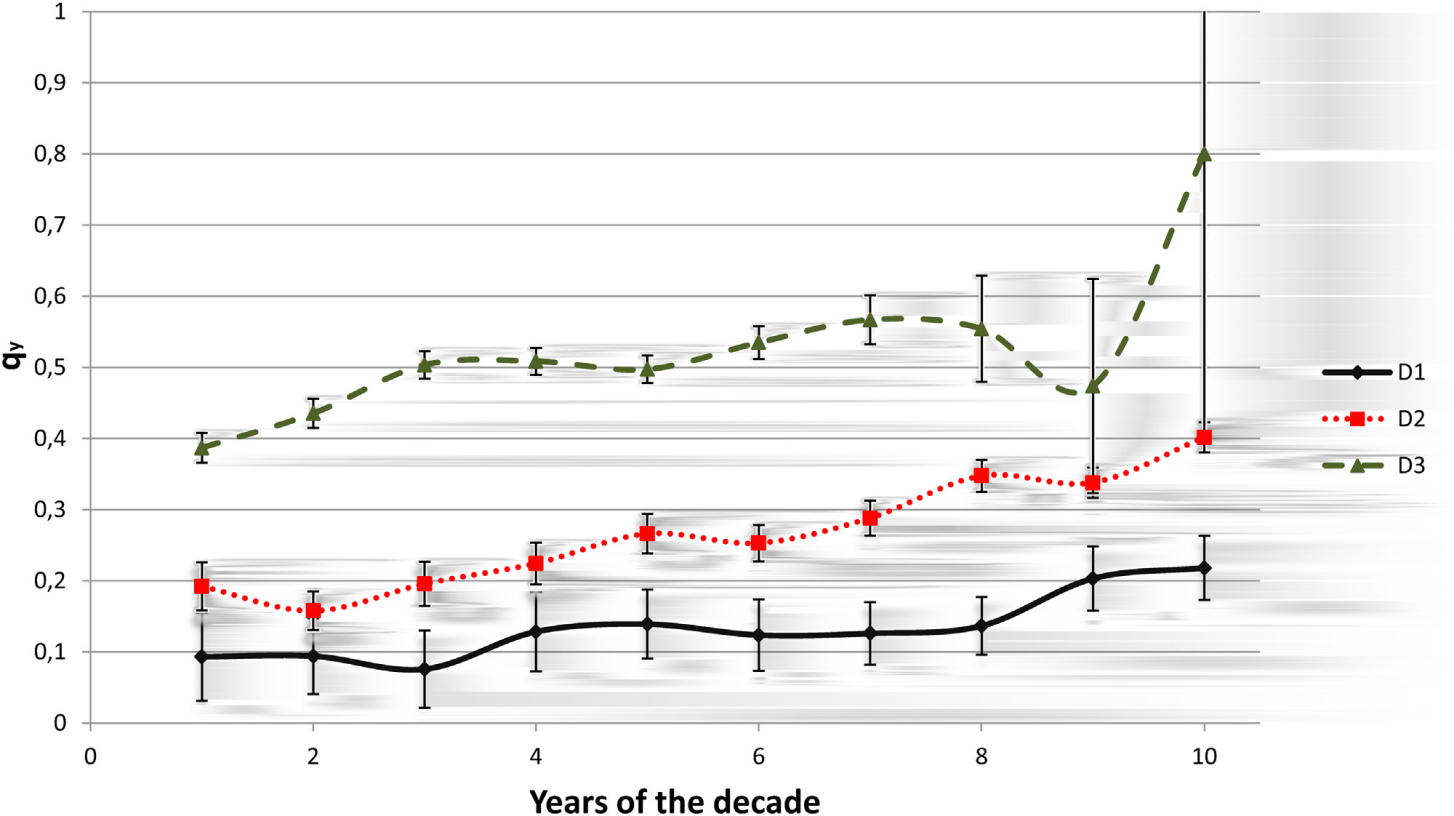
Proportion of A-A publications with regard to the total number of publications of the advisee (*q*_*y*_) for the Exact Sciences.

In analyzing the influence of the number of the A-A publications on the total number of the advisee’s publications, we have considered the proportion between these two measures. Over a period of decades, there was a continuous increase of this proportion (Fig 4) and a considerable difference between the decade D3 and the other two decades. This difference has shown statistical significance using the Mann-Whitney non-parametric test, the obtained statistics are W = 55 for both pairs (D1,D3) and (D2,D3) all with *p* < 0.01. This test has been used after verified the similarity of variance with the *F* and Levene tests with *p* > 0.1. The singular behavior and high degree of variability of this proportion in the last years of the decade D3 curve is due to the fact that exist a small number of researchers involved that began theirs graduation in this years and concluded it before 2010 or that some researchers do not update their curricula frequently [31]. The standard deviations are shown in Fig 4 and are up to 0.31, 0.04 and 0.4 for decades D1, D2 and D3, respectively.

We also noted that for the decade D3, this proportion for Chemistry, and Physics is greater than for the whole group of the Exact Sciences and for the other subareas this measure is less than that of the Exact Sciences (data not shown).

For the entire period, it is worth noting that the maximum of A-A publications was 158 and the mean average of the joint publications for Astronomy, Chemistry and Physics is greater than 4.6 and for the other subareas is less than 2.2. It is reasonable to assume that a small proportion of pairs that do not produce A-A works in these subareas can be correlated in an opposite way to the high mean average of the A-A publications. The mean of the number of advisee publications for the Exact Sciences is 11.39, with a standard deviation of 20.58, and the mean of the number of advisor publications is 51.78 with a standard deviation of 50.14. It has been noted that Physics is the subarea where advisors and advisees publish more than other subareas, followed by Chemistry and Astronomy. Mathematics and Computer Science are the subareas with the lowest number of journal publications.

### Measuring the time relationship versus research output

#### Correlation analysis

The Spearman correlation coefficient [32] was used to calculate the relationship between $T_{i}^{R}$ and *p*_*i*_ for the entire period for the Exact Sciences and their respective subareas. The correlation r = 0.424 was obtained for the Exact Sciences with *p* < 0.001. For all the subareas, this coefficient is higher than 0.34 with p-values less than 0.01, except for Computer Science (with r = 0.26 and p-value < 0.01). The highest r corresponds to Astronomy (r = 0.642 and p-value also less than 0.01).

Additionally, the Spearman correlation was calculated to assess the same variables by considering the decades when the relationship started. Moreover, as we noted in Chemistry and Physics, this measure was significant for all the decades (*p* < 0.01).

As shown in [25, 28], it was also found that there is a significant correlation between the total number of publications of the advisors and advisees. For all pairs in Exact Sciences that have published at least one output, it has been computed the Spearman correlation (r) from the first A-A publication. This value is 0.584 with *p* < 0.01.

#### Measuring the A-A relationship

All the measurements used in previous sections are related to the global behavior of the Exact Science area and their subareas. This static approach does not allow to analyze the dependence between the advisor and the advisee. To measure this influence, we have employed a modified version of the Kulczynski index that has been used to determine the dependence relationship between A-A.

We have analyzed the advisor-advisee relationship through time by adopting two approaches: the first took account of the entire period and the second separated it into decades setting out from the year of the first output $y_{ij}^{f}$. The novel feature of our approach is that it employs the Kulczynski index $kulc_{ij}^{t}$ to measure this relationship every year for each pair, until the year of the last A-A publication ($y_{ij}^{l}$). It should be highlighted that high values of $kulc_{ij}^{t}$ does not indicate high production; this will be explained in the Computer Science case.

**Fig 5:**
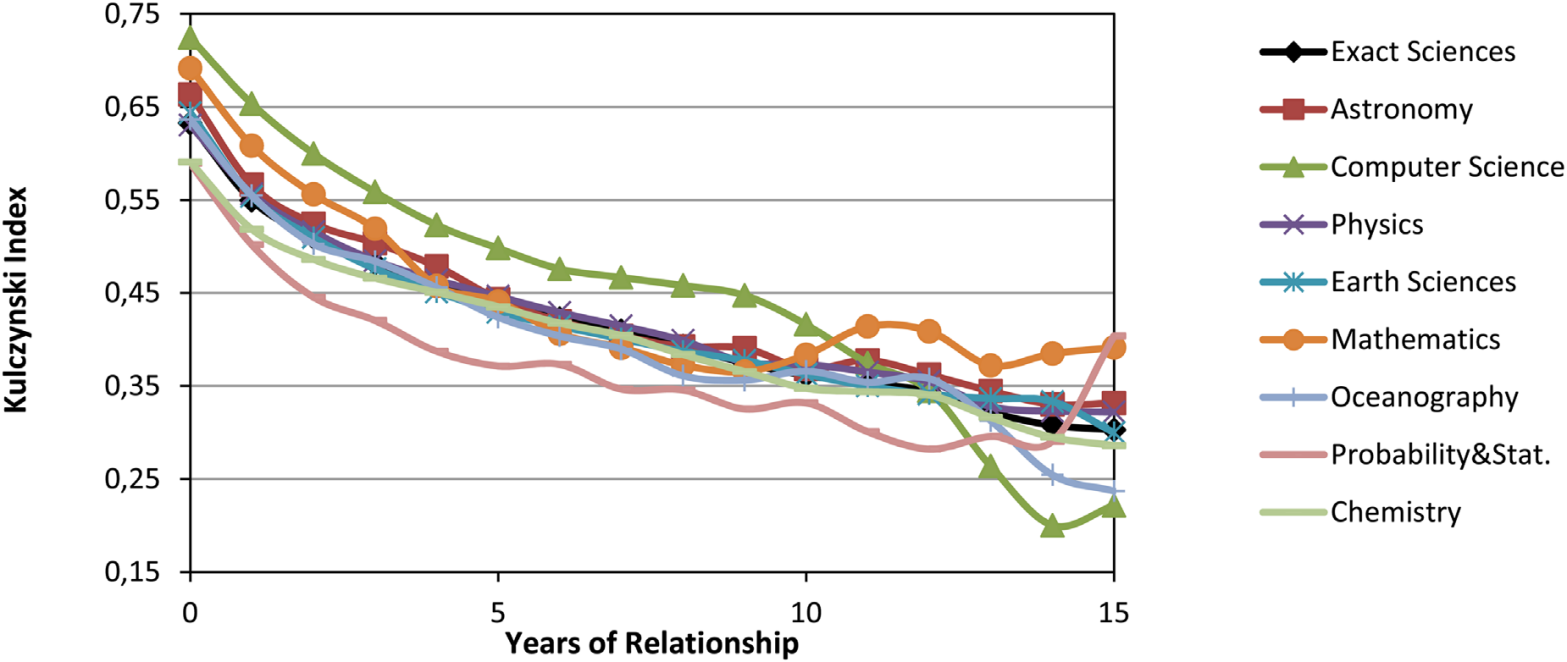
Mean of the Kulczynski index for Exact Sciences and all the subareas.

In the first approach, the mean and standard deviation of $kulc_{ij}^{t}$ was calculated for every year for the Exact Sciences and its eight subareas. Due to the large number of data, we have only shown the mean in Fig 5. For this period, the maximum value of the standard deviation is 0.22 for Oceanography in the first year, and the minimum is 0.11 for Computer Science in the last year. In Fig 5, it can be seen that the relationship declines gradually for every subarea. Probability & Statistics has the lowest index in most of the years of the analyzed period, which means that in Probability & Statistics the A-A dependence is fewer than in the other subareas for these years. It is worth noting that in the first ten years of the relationship in Computer Science, most of the advisees’ publications are produced jointly with their advisors which indicates high values in the Kulczynski index; however this does not imply that there are a large number of advisee’s publications.

**Fig 6:**
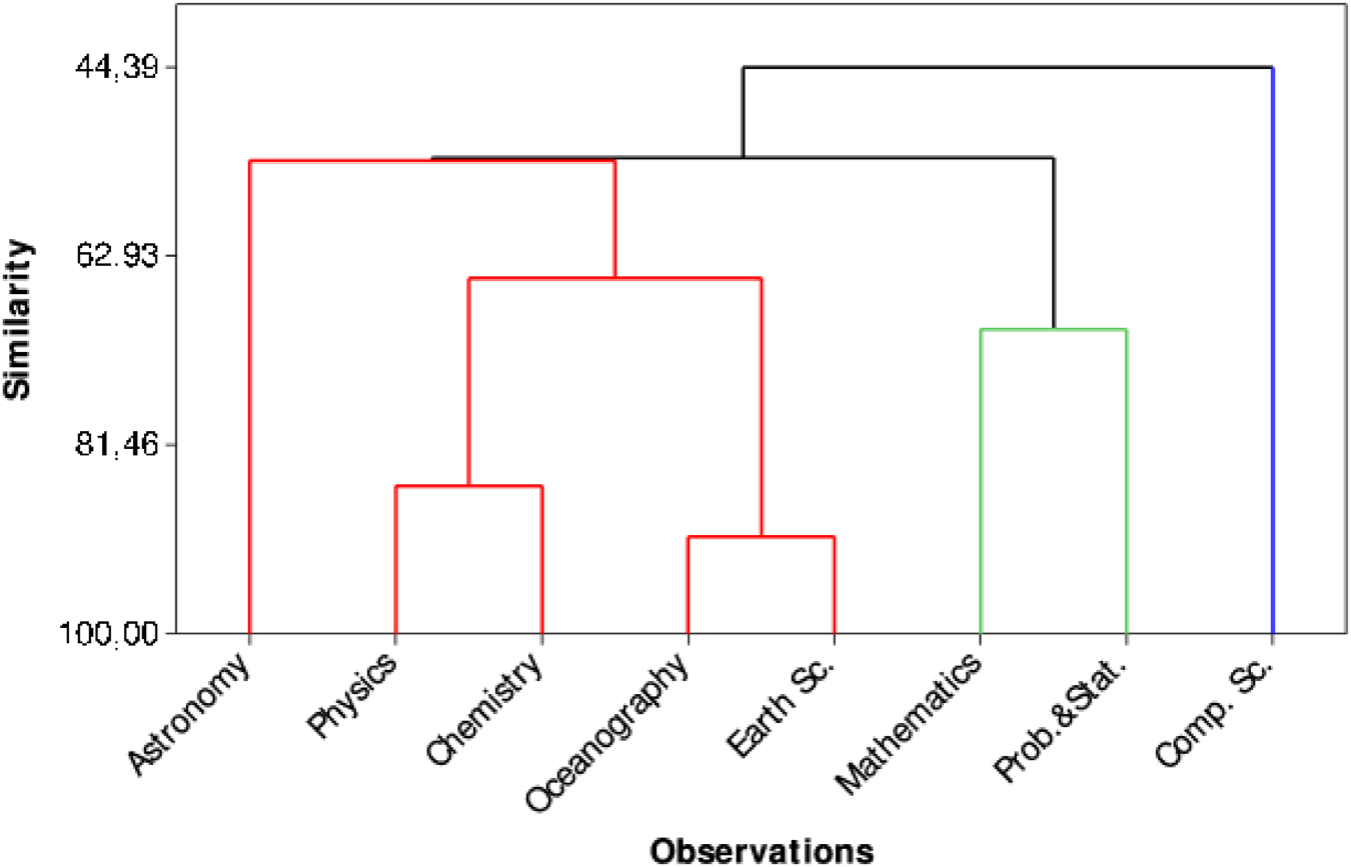
Dendrogram displaying the decline of the Kulczynski index until the 5th year.

In the second approach, we have constructed a dendrogram (Fig 6) of the mean of the Kulczynski index until the fifth year to detect distinguishing behavioral features in the subareas. Five clusters of subareas can be seen: (i) Astronomy, (ii) Physics and Chemistry, (iii) Oceanography and Earth Sciences, (iv) Mathematics and Probability & Statistics, and (v) Computer Science.

### Assessing the clusters of subareas

**Fig 7:**
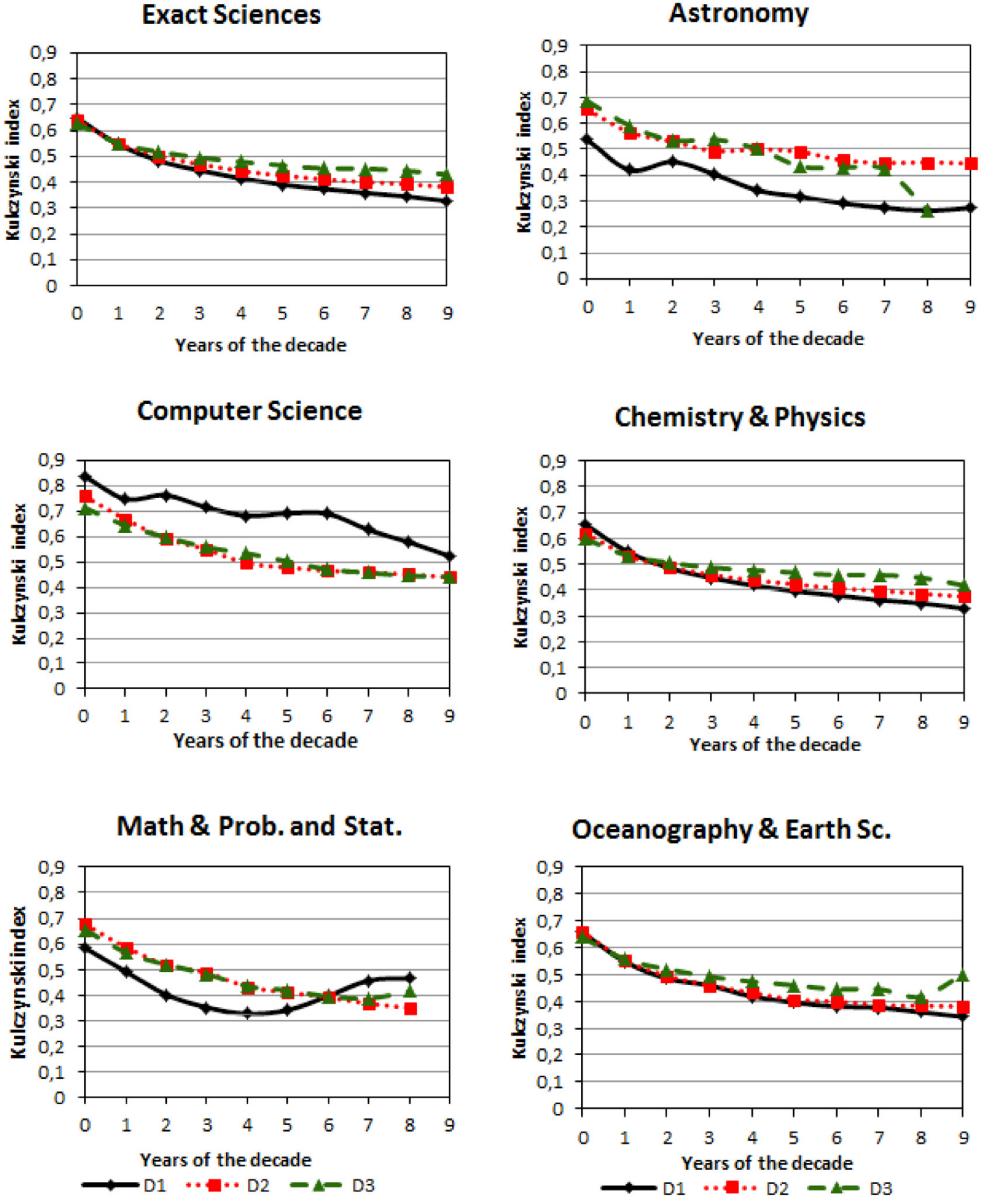
Mean of the Kulczynski index for decade D1, decade D2 and decade D3 for the Exact Sciences and the five clusters per subareas (according to the dendrogram).

In Fig 7, the mean average of the downward trend of the index for the Exact Sciences was compared with each cluster until the tenth year for each decade. It has been computed the standard deviation for each year of each decade. For the decade D1, the maximum value of the standard deviation (0.33) corresponds to the third year of Computer Science and the minimum (0.10) for the ninth year of Astronomy. For the decade D2, the maximum value is 0.19 for the second year of Mathematics & Statistics and the minimum 0.15 for the eighth year of Chemistry & Physics. Fort decade D3, the maximum value of the standard deviation (0.22) corresponds to second year of Mathematics & Statistics and the minimum (0.10) for the eighth year of Astronomy. In general, it has been observed that the standard deviations are similar for each decade.

In the Exact Sciences, it was found that for the decade D1 the Kulczynski index declined by a mean average of 35.48% until the fifth year and 48.38% until the tenth year. In the decade D2 the index decreased by a mean average of 31.25% until the fifth year and 40.63% until the tenth year, for the same periods, for D3 the index decreased by 25% and 34.38% respectively. It can be observed that in the last decade, the A-A dependence fell by a lower proportion than in the other decades. This result could be explained by the new Brazilian scientific policies that require more publications to be issued through partnerships in the early career.

In Fig 7, the relationship between the trend and the groups of decades of the Exact Sciences remains in two clusters (i) Physics and Chemistry, and (ii) Oceanography and Earth Sciences. In the other clusters, the groups have the same downward trend, but not the same relationship between the three decades. It is worth noticing that while the A-A dependence has fallen from D1 to the other decades for most of clusters; for the Computer Science cluster, the relationship between the decades is the opposite.

**Fig 8:**
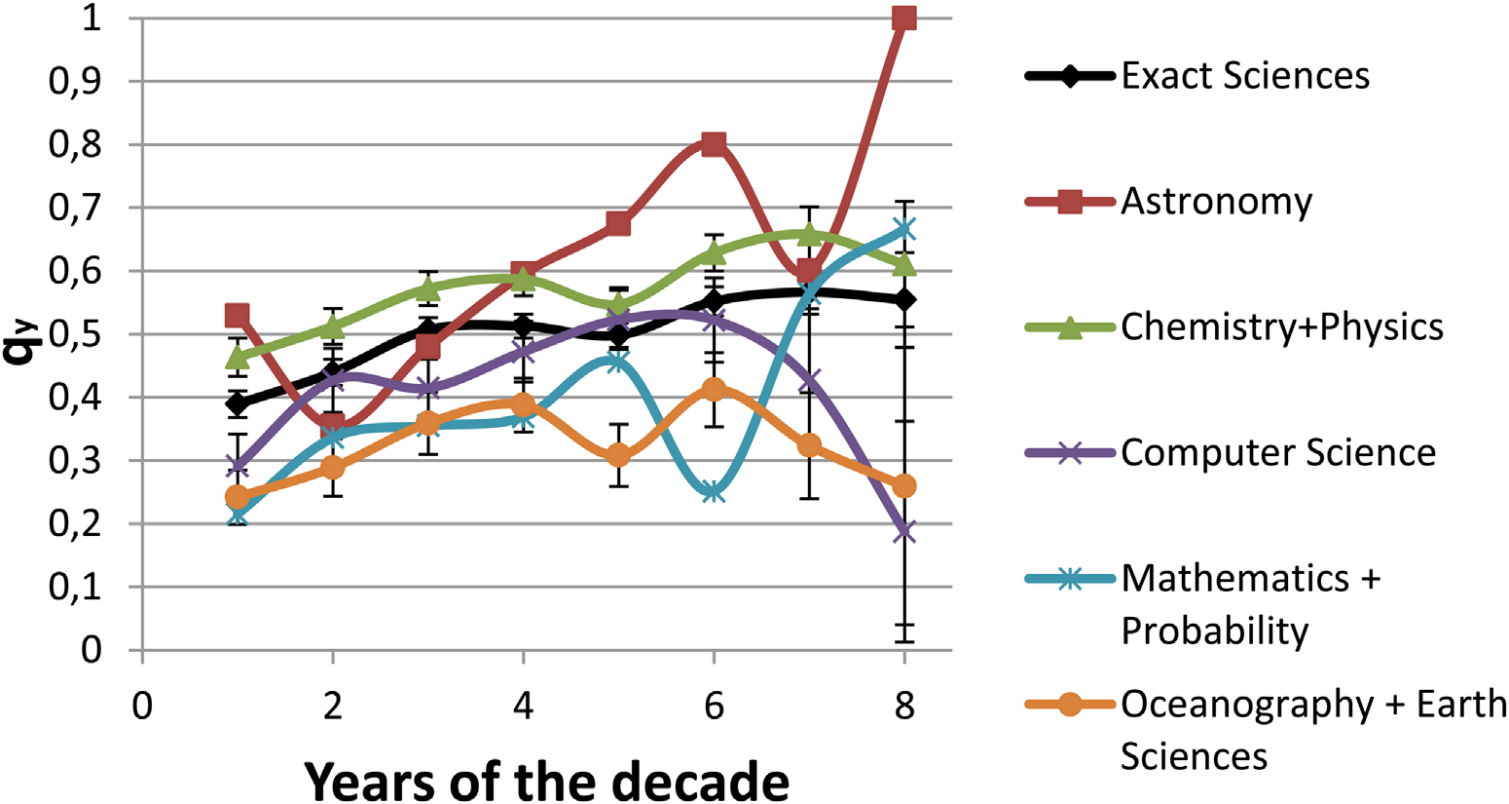
Proportion of A-A publications with regard to the publications of the advisee (*q*_*y*_) for decade D3 for the Exact Sciences and the five clusters per subareas (according to the dendrogram).

Fig 8 shows the proportion of A-A publications with respect to the total number of advisee’s publications (i.e. *q*_*y*_) for the five clusters for the decade D3 (the last two years have not been represented because the high variability of the proportions due to the fact that exist a small number of researchers involved). It can be seen that *q*_*y*_ for Chemistry and Physics is statistically significant greater than the Exact Sciences computed by the Mann-Whitney test with W = 87 and p = 0.026 in contrast the *q*_*y*_ for Oceanography & Earth Sciences and Computer Science is smaller than that Exact Sciences, with *W* = 85 (*p* = 0.041) and *W* = 99 (*p* = < 0.01) respectively.

Following, it is discussed the behavior of each of the five clusters.

#### Astronomy

Since there are few universities in Brazil that have Astronomy as an undergraduate course and most of researchers in Astronomy did Physics as their undergraduate course and then continued their scientific career in Astronomy, it is expected that the behavior of this cluster will be similar to the Physics and Chemistry cluster. However, in some respects, it is dissimilar due to the small number of researchers involved in this area, especially in decade D1. It can be seen that over a number of years, Astronomy has experienced a significant growth.

#### Chemistry and Physics

There has been a long tradition of publications with multiple authors in natural science, such as Chemistry and Physics, because of their need to work in large groups [25]. Nevertheless, it is possible that some joint publications might not necessarily have been the result of the doctoral work of the advisee. This could be one of the reasons why this cluster and the Astronomy cluster have achieved the lowest percentage of pairs who have never published jointly. It should be underlined that Chemistry and Physics are the subareas where traditionally pairs have always been committed to produce joint studies. This cluster has the lowest elapsed time for the first A-A publication. Moreover, this cluster shows a significant reduction in the percentage of pairs that have not publised from the decade D1 to D3. The period of the collaboration $T_{i}^{R}$ for this cluster is greater in about one year than the other subareas (in the mean and the median). This cluster and the Astronomy cluster show a shorter time to achieve the first publication with respect to the year of the PhD graduation (*z*_*i*_). Moreover, from decade D1 to D3 there is a significant reduction of *z*_*i*_ from the end of the doctoral period in D1 (*z*_*i*_ ≃ 0) to two years before it in decade D3 (*z*_*i*_ ≃ −2). Chemistry and Physics is a cluster where advisors and advisees publish more, the mean of the joint publications (*p*_*ij*_) is higher and the proportion of the joint publications with regard to the total number of publications of the advisees (*q*_*y*_) is greater than the other subareas. This implies a close relationship, but not a strong interdependence between the pairs (the Kulczynski index is neither the highest nor the lowest).

#### Computer Science

The publication culture in Computer Science has been different from the other subareas [33]. It is known that although works published in journals have a greater impact, the standard practice is to publish more in conferences [34, 35], and, sometimes, after publishing a conference paper, an extended version is published in a journal [33]. A conference provides an opportunity for fast and regular publications, as well as to meet other researchers and form new partnerships. In Computer Science, having a paper accepted at a leading conference is prestigious and also allows the work to be disseminated [33]. These characteristics are important for Computer Science since it is a relatively young and rapidly evolving subarea [34]. Currently, there is a debate about whether it is necessary to change the procedure of publishing a paper in a journal and the role of the conference in this subarea. As expected, advisors and advisees in Computer Science prefer to publish in conferences rather than in journals. This could be a reason for the results obtained for Computer Science with regard to the number of publications in journals. As was shown in our results, Computer Science has one of the lowest numbers of journal publications. In Brazil, this has not been a drawback because the graduate program assessment committee in Computer Science looks favorably on conference publications. It should be noted that this attitude in Computer Science is changing since researchers are required by the government agencies to publish in journals as well. This could be the reason for the significant reduction in the percentage of advisees who have not published with their advisors during the decade from D1 to D3. The government agencies also encourage independent research. Although Computer Science has higher values in the Kulczynski index in the decade D1 than the other clusters, the dependence between the advisor-advisee pair has declined from decade D1 to the other decades, which shows that it is a source of great concern in this cluster.

#### Mathematics and Probability & Statistics

Mathematics has traditionally been a domain of individual scientists rather than of teams [36]; however in this subarea there has been an increasing number of joint works. In the 40’s, 90% of math papers were single authored, but this percentage fell to about 50% in the last decade [37]. Despite this growth in the number of collaborations, it should be stressed that currently Mathematics has the lowest number of joint publications among all the subareas [32, 36, 38]. [32, 39] have explained this phenomenon by suggesting that theoretical studies can be produced with fewer authors than experimental work. However, Mathematics still has the smallest number of journal publications and the highest percentage of advisees without joint publications. Although Mathematics has shown the longest time for the first publication (*z*_*i*_), this situation has been improving considerably in recent years. In the decade D1, the mean average of this time was 4 years after the finish of the PhD (*z*_*i*_ ≃ 4) and in the decade D3 it changed to the same period as the end of the doctorate (*z*_*i*_ ≃ 0). Probability & Statistics has the lowest values in the Kulczynski index in most of the years of the analyzed period and also less proportions of joint publications with regard to the total number of publications of the advisee (*q*_*y*_) than Exact Sciences in most of the years. However, the percentage of advisees without joint publications is not one of the greatest and is about 35%. This means that the advisees are independent, and the number of advisee’s publications that have been issued with their respective advisors are fewer than in the other subareas for these years.

#### Oceanography and Earth Sciences

There are few works that investigate the scientific output of the area of Oceanography in Brazil. This area includes a low number of researchers and thus a low number of pairs (Table 1). It is also the area with the lowest number of A-A publications. One of the factors that may have caused the low number of researchers is the recent consolidation of Oceanography in the country [40]. In [40] the areas of Astronomy and Oceanography are compared, which suggests that Astronomy publications differ from Oceanography in term of the international attention they receive and also that the most cited publications in Oceanography are geared towards the national interest. The other area in this cluster, Earth Sciences, seems to be different from Oceanography in terms of the number of researchers (Table 1). However, in our study, in terms of the interdependence between A-A, Oceanography and Earth Science are one of the pair areas that are most closely related, according to Fig 6. One possible reason for this is that in some universities of Brazil, Oceanography is treated as a part of Earth Science. The behavioral patterns in the A-A relationship, when almost all the metrics assessed in this study for Oceanography and Earth Science clusters are taken into account, is similar to the behavior displayed in Fig 8, where the proportion of joint publications with regard to the total number of publications of the advisee (*q*_*y*_) for this cluster is smaller than the other clusters with the statistics *W* between 87 and 100 and *p* < 0.026 computed by the Mann-Whitney test except for Mathematics and Probability & Statistics with *W* = 77 and *p* = 0.372.

## Conclusion

In this paper, we have sought to outline some features of the time relationship and publications between advisors and advisees registered in the Exact and Earth Sciences Area of the Lattes platform from 1981 to 2010. To the best of our knowledge, this is the first study in large scale of the relationship between advisors and advisees (8,265 pairs). The percentage of pairs that never published was 28%. For the other set of pairs, different from Pinheiro et al (2014), in this work we have found evidence that the time of PhD training to develop skills and “know-how” is not limited to the formal period of supervision, but it could extrapolate it. Additionally, we detected a positive and significant correlation between the duration of the relationship and the number of advisee publications, as measured by the Spearman Correlation Coefficient. This means that the duration of the relationship correlates positively with the scientific productivity of the advisee (measured by the number of journal papers published).

Nevertheless, the evolution of the relationship between the pairs measured by the Kulczynski index decline throughout the time period under study. This suggests that the PhD graduate gets more independence with respect to his advisor along the years and/or increments his research collaboration group.

## References

[1] de Meis L, Arruda AP, Guimaraes J. The impact of science in Brazil. IUBMB Life. 2007;59(4–5):227–234. 10.1080/15216540701258140 17505957

[2] Coutinho RX, Dávila ES, dos Santos WM, Rocha JaBT, Souza DOG, Folmer V, et al Brazilian scientific production in science education. Scientometrics. 2012;92(3):697–710. 10.1007/s11192-012-0645-5

[3] Bin A, Salles-Filho S, Capanema LM, Colugnati FAB. What difference does it make? Impact of peer-reviewed scholarships on scientific production. Scientometrics. 2014;online(1):1–22.

[4] Pinheiro D, Melkers J, Youtie J. Learning to play the game: Student publishing as an indicator of future scholarly success. Technological Forecasting & Social Change. 2014;81:56–66. 10.1016/j.techfore.2012.09.008

[5] Igami MPZ, Bressiani JC, Mugnaini R. A new model to identify the productivity of theses in terms of articles using co-word analysis. Journal of Scientometric Research. 2014;3:3–14.

[6] Salmi LR, Gana S, Mouillet E. Publication pattern of medical theses, France 1993–98. Medical Education. 2001;35(1):18–21. 10.1046/j.1365-2923.2001.00768.x 11123590

[7] Frkovic V, Skender T, Dojcinovic B. Publishing scientific papers based on Master’s and Ph.D. theses from a small community: case study Croatian medical schools. Croatian Medical Journal. 2003;44(1):107–111. 12590439

[8] Ramos PS, Furtado EC, Carvalho ERF, Campos MO, Cortes de Souza DVB, Almeida LDP, et al Dissertações e Teses de Pós Graduação geram publicação de artigos científicos? Análise baseada em 3 programas da área de educação física. Brazilian Journal Biomotricity. 2009;3(4):315–324.

[9] Sacardo MS, Hayashi MCPI. Balanço bibliométrico da produção científica em Educação Física e Educação Especial oriunda de teses e dissertações. RBPG Revista Brasileira de Pós-Graduação. 2011;8(15):111–135.

[10] Arriola-Quiroz I, Curioso WH, Cruz-Encarnacion M, Gayoso O. Characteristics and publication pattern of theses from a Peruvian medical school. Health Information and Libraries Journal. 2010;27:148–154. 10.1111/j.1471-1842.2010.00878.x 20565556

[11] Anwar MA. From doctoral dissertation to publication. A study of 1995 American graduates in library and information science. Journal of Librarian and Information Science. 2004;36 (4):151–157. 10.1177/0961000604050565

[12] Lee WM. Publication trends of doctoral students in three fields from 1965–1995. Journal of the American Society for Information Science. 2000;51(2):139–144. 10.1002/(SICI)1097-4571(2000)51:2<139::AID-ASI5>3.0.CO;2-1

[13] Mallette LA. Publishing rates of graduates education Ph.D. and Ed.D. students: a longitudinal study of University of California schools. Doctoral dissertation, Pepperdine University Retrieved from ProQuest Dissertations & Theses database (UMI No 3239922). 2006;.

[14] Sayers MK, Wood FE. The use and value of MSc Information Studies dissertations. Journal of Information Science. 1991;17(5):307–314. 10.1177/016555159101700507

[15] Larivière V. On the shoulders of students? The contribution of PhD students to the advancement of knowledge. Scientometrics. 2012;90(2):463–481. 10.1007/s11192-011-0495-6

[16] Fonseca L, Velloso S, Wofchuk S, Meis L. The relationship between advisors and students. Scientometrics. 1998;41(3):299–312. 10.1007/BF02459047

[17] Mainhard T, van der Rijst R, van Tartwijk J, Wubbels T. A model for the supervisor doctoral student relationship. Higher Education. 2009;58(3):359–373. 10.1007/s10734-009-9199-8

[18] Golde CM. Should I stay or should I go? Student descriptions of the doctoral attrition process. Review of Higher Education. 2000;23(2):199–227. 10.1353/rhe.2000.0004

[19] Kam BH. Style and quality in research supervision: the supervisor dependency factor. Higher Education. 1997;34(1):81–103. 10.1023/A:1002946922952

[20] Marsh HW, Rowe KJ, Martin A. Ph.D. Students? Evaluations of Research Supervision. Higher Education. 2002;73(3):313–348. 10.1353/jhe.2002.0028

[21] McAlpine L, Norton J. Reframing our approach to doctoral programs: an integrative framework for action and research. Higher Education Research and Development. 2006;25(1):3–17. 10.1080/07294360500453012

[22] Kamler B. Rethinking doctoral publication practices: Writing from and beyond the thesis. Studies in Higher Education. 2008;33(3):283–294. 10.1080/03075070802049236

[23] Netting FE, Nichols-Casebolt A. Authorship and collaboration. Journal of Social Work Education. 1997;33(3):555–564.

[24] Goldsmith SS, Presley JB, Cooley EA. National Science Foundation Graduate Research Fellowship Program; 2002 Final Evaluation Report. Virginia: NSF.

[25] Wang C, Han J, Jia Y, Tang J, Zhang D, Yu Y, et al. Mining advisor-advisee relationships from research publication networks. In: Knowledge Data Discovery (KDD); 2010. p. 203–212.

[26] Wang C, Han J, Li Q, Li X, Lin WP, Ji H. Learning Hierarchical Relationships among Partially Ordered Objects with Heterogeneous Attributes and Links. In; Proceedings of 2012 SIAM International Conference on Data Mining; 2012. p.516–527.

[27] Wu T, Chen Y, Han J. Association Mining in Large Databases: A Re-examination of Its Measures. In: Knowledge Discovery in Databases: PKDD; 2007 p. 621–628.

[28] Wu T, Chen Y, Han J. Re-examination of interestingness measures in pattern mining: a unified framework. Data Mining and Knowledge Discovery. 2010;21(3):371–397. 10.1007/s10618-009-0161-2

[29] Mena-Chalco JP, Digiampietri LA, Lopes FM, Cesar RM. Brazilian bibliometric coauthorship networks. Journal of the Association for Information Science and Technology. 2014;65(7):1424–1445. 10.1002/asi.23010

[30] Digiampietri LA, Mena-Chalco JP, Vaz de Melo POS, Malheiro APR, Meira DNO, Franco LF, et al BraX-Ray: An X-Ray of the Brazilian Computer science Graduate Programs. PLoS ONE. 2014 04;9(4):e94541 10.1371/journal.pone.0094541 24728179 PMC3984164

[31] Digiampietri LA, Mugnaini R, Mena-Chalco JP, Delgado KV, Alcazar JJP. Análise macro das últimas atualizações dos Currículos Lattes. Em Questão Edição Especial. 2014;20(3):88–113.

[32] Myers JL, Well AD. Research Design and Statistical Analysis. 2nd ed Lawrence Erlbaum; 2003.

[33] Halpern J, Parkes D. Journals for certification, conferences for rapid dissemination. Communications of the ACM. 2011;54(8):36–38. 10.1145/1978542.1978555

[34] Franceschet M. The role of conference publications in Computer Science: a bibliometric view. Communications of the ACM. 2010;53(12):129–132. 10.1145/1859204.1859234

[35] Vardi M. Conferences vs. Journals in Computing Research. Communications of the ACM. 2009;52(15).

[36] Glänzel W, Schubert A. Analysing scientific networks through coauthorship Handbook of quantitative science and technology research. Kluwer Academic Publishers; 2004 p. 257–276.

[37] Struppa DC. The New Publishing Scene and the Tenure Case: An Administrator’s View. Notice of the AMS. 2012;59(5):670–671.

[38] Mena-Chalco J, Cesar-Jr R. Towards automatic discovery of co-authorship networks in the Brazilian academic areas. In: IEEE Seventh International Conference on e-Science; 2011. p. 53–60.

[39] de Souza Vanz SA, Stumpf IRC. Colaboração científica: revisão teórico conceitual. Perspectivas em Ciência da Informação. 2010;15(2):42–55.

[40] Leta J. Human resources and scientific output in Brazilian science: Mapping astronomy, immunology and oceanography. Aslib Proceedings. 2005;57(3):217–231. 10.1108/00012530510599181

